# Boolean Networks with Classic and New Updating Modes Applied to Genetic Regulation in Some Familial Diseases

**DOI:** 10.3390/ijms262411976

**Published:** 2025-12-12

**Authors:** Jacques Demongeot, Abdoul Khadir Diallo, Hana Hazgui, Mariem Jelassi, Fatine Kelloufi, Houssem ben Khalfallah, Alonso Espinoza, Marco Montalva-Medel

**Affiliations:** 1 Laboratory AGEIS, Faculté de Médecine, Université Grenoble Alpes, Avenue des Maquis du Graisivaudan, 38700 La Tronche, France; abdoul-khadir.diallo@etu.univ-grenoble-alpes.fr (A.K.D.); fatine.kelloufi@etu.univ-grenoble-alpes.fr (F.K.); houssem.ben-khalfallah@univ-grenoble-alpes.fr (H.b.K.); 2 CHS Montperrin, Assistance Publique Hôpitaux de Marseille, 109 Avenue du Petit Barthélémy, 13090 Aix-en-Provence, France; hana.hazgui@ch-montperrin.fr; 3 ENSI-Ecole Nationale des Sciences de l’Informatique, Campus Universitaire de la Manouba, La Manouba 2010, Tunisia; mariem.jelassi@ensi-uma.tn; 4 Faculty of Engineering and Science, Universidad Adolfo Ibáñez, Av. Diagonal Las Torres 2640, Peñalolén 7910133, Santiago, Chile; alonespinoza@alumnos.uai.cl (A.E.); marco.montalva@uai.cl (M.M.-M.)

**Keywords:** Boolean network, genetic regulatory network, structural robustness, gene interaction graph, familial angioedema, osteogenesis imperfecta, biliary atresia

## Abstract

Many familial diseases are caused by genetic accidents, which affect the genome and its epigenetic environment, summarized as an interaction network between genes. We wish to study the existence or absence of robustness for such genetic interaction networks centered on the gene SP1 and involved in three familial diseases: familial angioedema, osteogenesis imperfecta, and biliary atresia. The updating of gene states at the vertices of the interaction graph of the genetic network (1 if a gene is activated, 0 if it is inhibited) can be performed in multiple ways that have been well-studied over the last 20 years: parallel, block-parallel, sequential, block-sequential, random, etc. We add to these classic updating modes two new ones, the intricate and the state-dependent. We have studied the robustness of three particular interaction graphs related to the familial diseases chosen as examples. The comparison of the interaction graphs and dynamics of the chosen familial diseases of different etiology shows common points in their interaction graphs and similarities in their dynamics according to their expression clock.

## 1. Introduction

**Figure 1:**
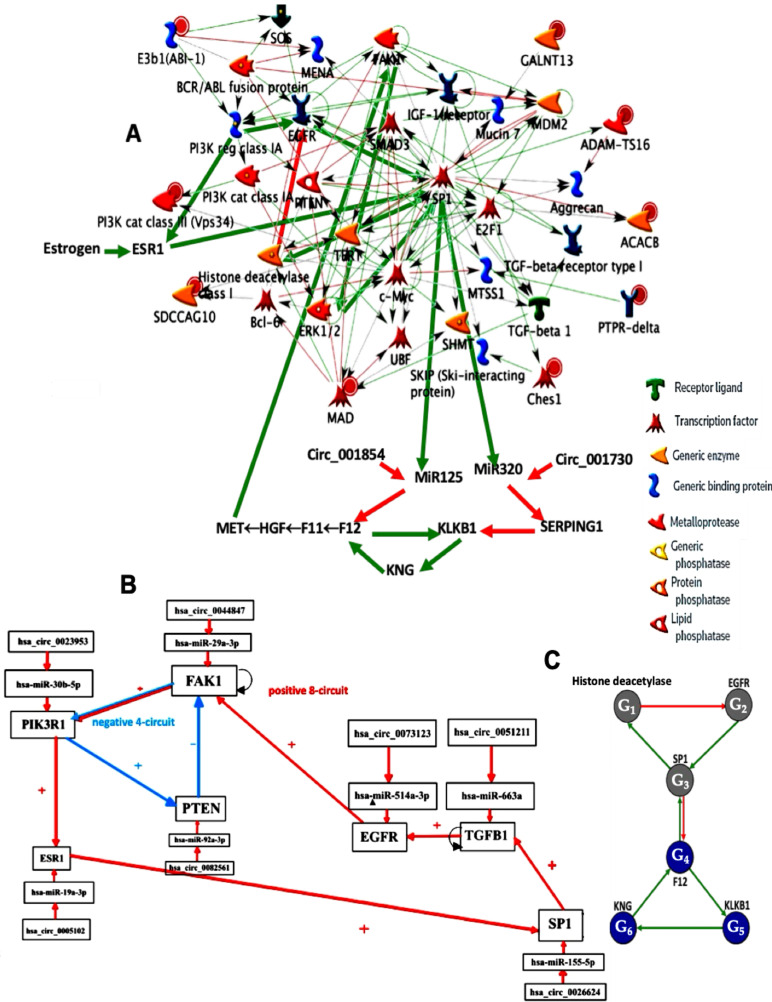
(**A**) Genetic network regulating the gene expression in familial angioedema, with red arrows for inhibitions and green arrows for activations. (**B**) Upper part of the network completed with control elements, i.e., specific microRNAs and circular RNAs. The negative circuit of length 4 is in blue, the positive circuit of length 8 in red. Self-activations were counted; (**C**) lower part of the regulation graph called SP1 subnetwork.

Many familial diseases are often caused by accidents occurring in the genome of the first pathological carrier in the family, due to abnormal endogenous rewriting of the genetic message during meiosis or to exogenous influences (irradiation, viral pathology, etc.). These accidents include, for example, point mutations that alter a single amino acid, or more significant chromosomal recombinations of the genome, such as translocations, causing radical changes in the interactions between the altered genes, their regulatory RNAs (siRNA, miRNA, circRNA, etc.), and the proteins belonging to their epigenetic regulatory network. Figure 1A shows the graph of these interactions in the case of a regulatory network centered on the ubiquitous transcription factor SP1, which plays a central role in many cellular pathways [1]: SP1 activates or represses the transcription of many other genes in response to physiological and pathological stimuli by binding, with high affinity, to GC-rich motifs in these genes involved in various processes such as apoptosis and cell growth, differentiation, immune response, cellular response to DNA damage, biological clock regulation, chromatin remodeling, response to oxidative stress, etc. Due to this ubiquitous regulatory role, SP1 is implicated in many familial diseases such as familial angioedema [2,3,4,5,6,7,8,9,10,11,12,13,14,15,16,17,18,19,20,21,22,23,24], osteogenesis imperfecta [25,26,27,28,29,30,31,32,33], and biliary atresia [34,35,36,37,38,39,40,41,42,43,44,45,46,47,48,49,50,51,52].

In [2,3,4,5,6], the role of the plasminogen activator PA is highlighted in the genesis and regulation of gene expression in hereditary angioedema, and its inhibitor PAI-1 is upregulated by TGF-β itself, which is activated by SP1. In [7,8,9,10,11,12,13,14,15,16,17,18,19], other genes involved in familial angioedema have been extensively studied, such as the angiopoietin-1 gene [7], the FXII gene [16], and SERPING1 [8,9,10,11,12,13,14,15,16,17,18,19]. The microRNAs inhibiting these genes were studied in [20] and the combined role of the Wnt and Notch pathways was presented in [21,22,23]. Finally, a clinical perspective on the importance of the combined role of all genes involved in familial angioedema can be found in [24,25]. In [25,26,27,28,29,30,31,32], it has been proven that TGF-β and its activator SP1 [26], the c-Met [27,28,29,30], sclerostin [31], Smad-3 [32], and MMP-13 [33] genes are involved in the genesis of osteogenesis imperfecta. Treatments for this familial disease take all these pathological factors into account [34]. In [35,36,37,38,39,40], numerous genes, such as those for fibrillin-1 [35,36], gamma-glutamyl transferase [37], interleukin-33 [38], and interleukin-8 [39], as well as SP1 and NFkB [40], have been identified in the causal mechanism of biliary atresia. Control by microRNAs [41] and the involvement of the elastin gene [42], Notch upregulated by SMAD3 and MDM2 [43,44,45], MMP-7 [46,47], and annexin genes [48] have also been identified. Taking this complex regulatory mechanism into account makes it possible to monitor gene expression profiles and adjust treatment for biliary atresia [49,50,51,52].

In the following, we study the attractors of Boolean genetic networks that regulate the main genes whose mutations cause these diseases. We identify the basins of attraction of these attractors and demonstrate their robustness to different types of perturbations, changes in initial conditions, and parameter values (modifying the interaction graph, transition function, or update clock) that can lead to three types of robustness loss: trajectory for initial conditions, asymptotic for final states, and structural for update modes. We will present the mathematical and numerical methods in Section 4 and some results in Section 2, then discuss these results in Section 3 and conclude in Section 5, with prospects for future robustness studies in the context of epigenetic regulation.

## 2. Results

The exact functioning of the chromatin clock controlling gene expression is still largely unknown. Therefore, the study of the robustness of genetic networks in the face of a change in initial or final conditions (trajectorial or asymptotic robustness) or a modification of their updating mode (structural robustness) is essential to understand the pathological behaviors linked to appearance in their dynamics of new characteristics such as the following:

- The enlargement or, on the contrary, the reduction in the size of their attractor basins (trajectorial robustness);
- The change in the nature of an attractor, for example, the transition from a stationary state to a limit cycle after the passage of a parameter value above a bifurcation threshold (asymptotic robustness);
- The birth of a new attractor, for example, following a modification of the updating rule (structural robustness).

As examples of application, three genetic regulatory networks are studied below. They are associated with three familial diseases: familial angioedema [2,3,4,5,6,7,8,9,10,11,12,13,14,15,16,17,18,19,20,21,22,23,24], osteogenesis imperfecta [25,26,27,28,29,30,31,32,33], and biliary atresia [34,35,36,37,38,39,40,41,42,43,44,45,46,47,48,49,50]. The gene regulatory networks corresponding to these diseases are described below. They contain genes and interactions from the literature [2,3,4,5,6,7,8,9,10,11,12,13,14,15,16,17,18,19,20,21,22,23,24,25,26,27,28,29,30,31,32,33,34,35,36,37,38,39,40,41,42,43,44,45,46,47,48,49,50] and reference databases [53,54,55,56]. Simulations of the relevant networks were carried out with the public software BoolNet [57] and by specific programs of network design and dynamical characteristics calculation [58,59].

### 2.1. Familial Angioedema

#### 2.1.1. Interaction Graph of the Familial Angioedema Genetic Network

Figure 1 illustrated the network regulating genes involved in angioedema, a familial disease with a prevalence of approximately 1 in 50,000, with two types of SERPING1 gene expression: low level (type I) and high level, but not correct functioning (type II). This network was provided by MetaCore^©^ [60] and subsequently verified in the literature.

#### 2.1.2. Familial Angioedema Network Dynamics

**Figure 2:**
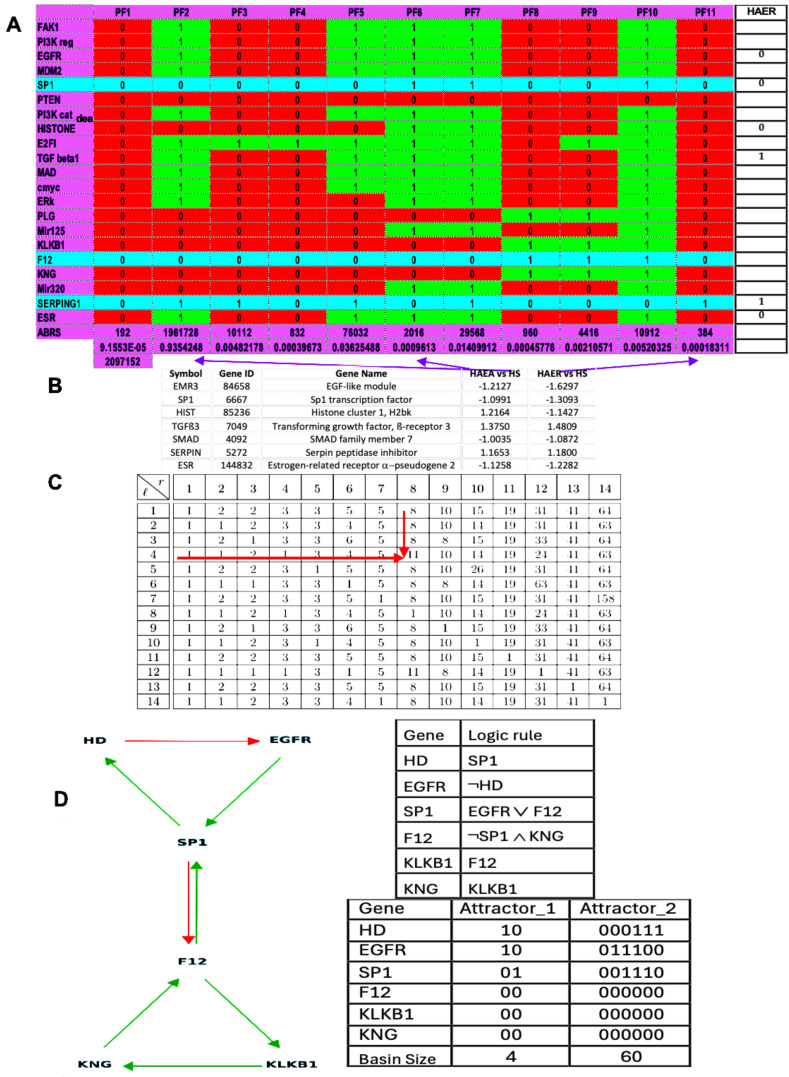
(**A**) Attractors from the simulation, in parallel mode, of the dynamics of the genetic network regulating the expression of the familial angiœdema (obtained using the software Network-Design^©^ [58]). (**B**) Expression data of angiœdema comparing the pathologic states to the healthy one. (**C**) Calculation of the attractor number of two tangent networks, one negative and of length *l* = 4 and the other positive and of length *r* = 8. (**D**) Attractors of the subnetwork SP1 (using in parallel mode the software BoolNet [57]).

Recent data on the mechanism of familial angioedema come from [2,3,4,5,6,7,8,9,10,11,12,13,14,15,16,17,18,19,20,21,22,23,24] and are given on Figure 2.

A previous study [57] predicted 11 attractors’ fixed points, both in theoretical and simulated results (Figure 2). The present results on Figure 2A,C are partly consistent with the expression data in Figure 2B: attractor PF6 could represent hereditary angioedema in acute state (HAEA) and attractor PF11 represents the remission state (HAER) despite the increased expression of TGFß. The main difference between HAE and HS (healthy state) is the decrease in the EGFR expression, especially in HAER, and the alternance of histone deacetylase between HAEA and HAER. This decrease and alternance exist both in parallel simulations of HAE attractors of the whole network as well as in the HAE attractor (limit cycle 1) of the subnetwork representing the core of the regulation (Figure 1C). The physiological attractor HS could correspond to the fixed point PF3 whose ABRS equals 93.43%, while ABRS of pathological PF6 and PF11 equals about 0.1%, percentages consistent with the observed prevalence of HAE in the general population, ranging from 1:10,000 to 1:150,000 [18,19]. Pathological attractor PF6 could correspond to the HAEA type with the absence of histone deacetylase expression [20]. When estrogens are present, they promote SP1 expression via ESR1 (see Figure 1A), which may explain why women are more numerous in type II angioedema and present more often an acute state than men.

Other attractors could only attract lethal or asymptomatic states. Lethal states are not observed due to their non-viability in utero and asymptomatic states could be revealed only during intercurrent illnesses or during aging processes.

### 2.2. Osteogenesis Imperfecta

#### 2.2.1. Interaction Graph of the Osteogenesis Imperfecta Genetic Network

**Figure 3:**
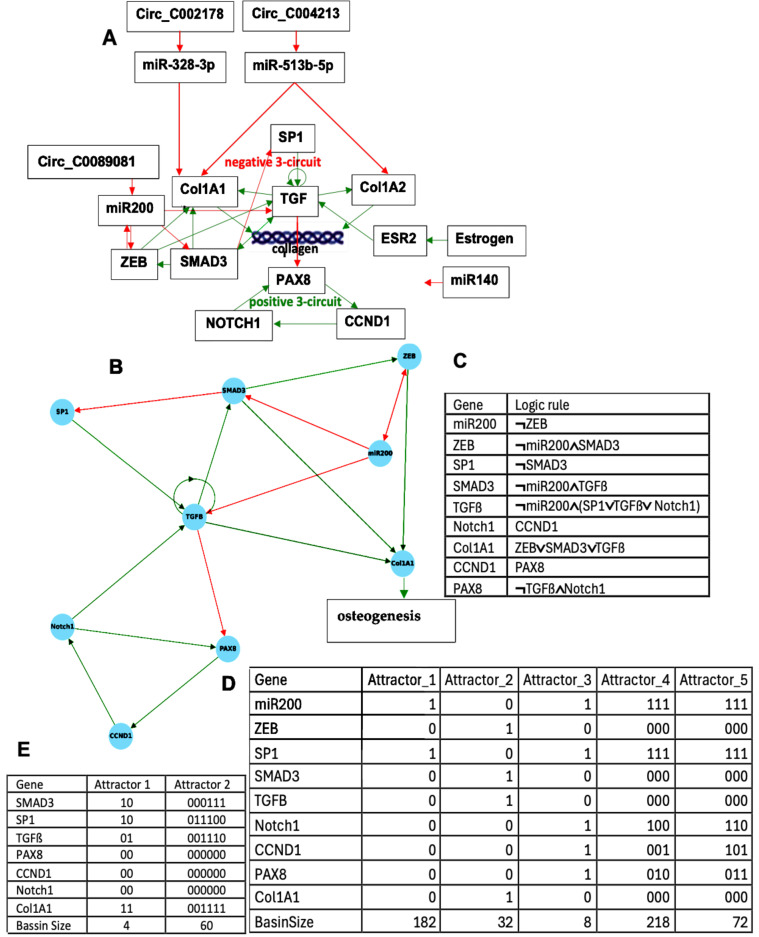
(**A**) Genetic network regulating the gene expression in osteogenesis imperfecta; (**B**) subnetwork TGFß representing the core of the regulation; (**C**) logic equations of (**B**); (**D**) attractors of subnetwork TGFß; and (**E**) attractors of subnetwork B without ZEB and miR200.

Figure 3 shows the network regulating the genes involved in osteogenesis imperfecta. All the interactions as Notch1 activating TGFß through M2 polarization come from the literature [25,26,27,28,29,30,31,32,33].

#### 2.2.2. Dynamics of the Osteogenesis Imperfecta Network

**Figure 4:**
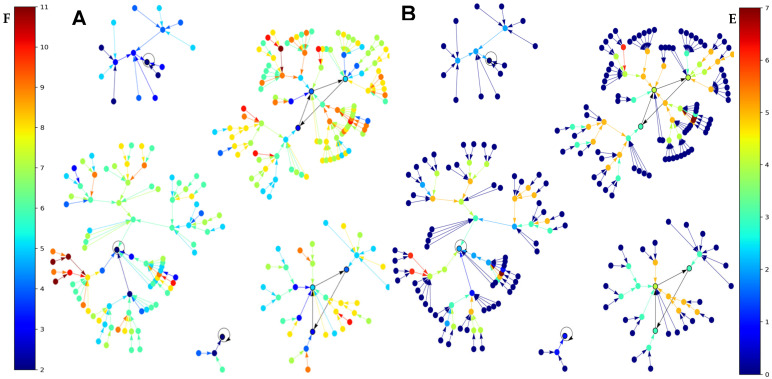
(**A**) Graph of the global frustration evolving from initial conditions to the five attractors of the subnetwork TGFß (without Col1A1) mainly decreasing along trajectories; (**B**) same graph for the global energy (using the software Espinoza^©^ [59]).

Results concerning the dynamics of a simplified version of the core of the network associated with familial osteogenesis imperfecta are given in Figure 3. Figure 3C presents the logical version of the transition function of the simplified interaction graph involved in osteogenesis imperfecta. By simulating, in parallel updating mode, the iterations of the Boolean network with this transition, we obtain (Figure 3D), from almost any initial configuration, four attractors with TGFß inactive, then a pathologic osteogenesis except for 32/512 = 6% of them leading to a fixed point (Figure 4).

This dynamical behavior is mainly due to the inhibition of TGFß by miR200. If miR200 is inhibited by the circular RNA Circ_C0089081, we can consider the network reduced to the subnetwork of Figure 3B without ZEB and miR200 having an attractor limit cycle of length 6 with a large basin of 60 configurations (ABRS = 94%), described in Figure 3E, with TGFß and Smad3 active together third-time, then allowing osteogenesis and thus corresponding to the physiological behavior.

If ABRS of the other limit cycle of length 2 equals 6% with TGFß and Smad3 never active together, then it could correspond to a pathologic behavior [32].

### 2.3. Biliary Atresia

#### 2.3.1. Interaction Graph of the Biliary Atresia Genetic Network

**Figure 5:**
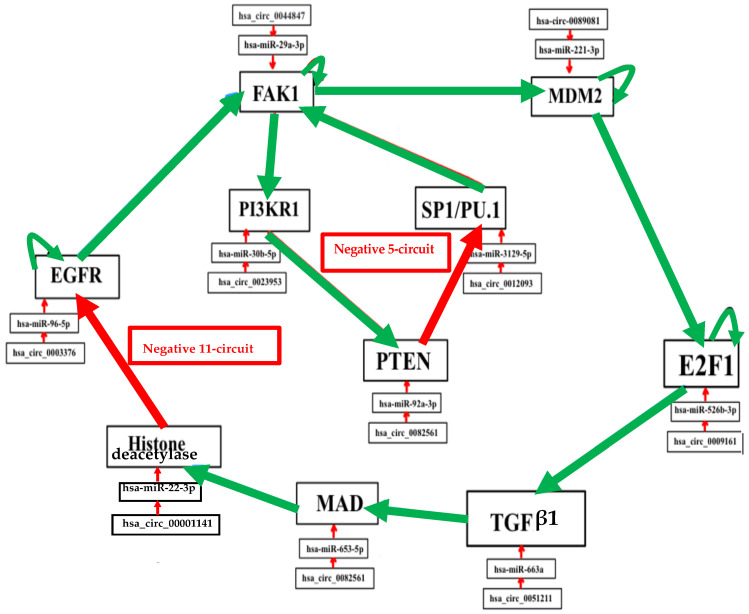
Genetic network regulating the expression of the biliary atresia with control elements, like specific microRNAs and circular RNAs. Negative interactions are represented by arrows in red and positive interactions are in green. Self-activations were counted.

Biliary atresia network of Figure 5 comes from the network of Figure 1A [35,36,37,38,39,40,41,42,43,44,45,46,47,48,49,50,51,52].

#### 2.3.2. Dynamics of the Biliary Atresia Network

**Figure 6:**
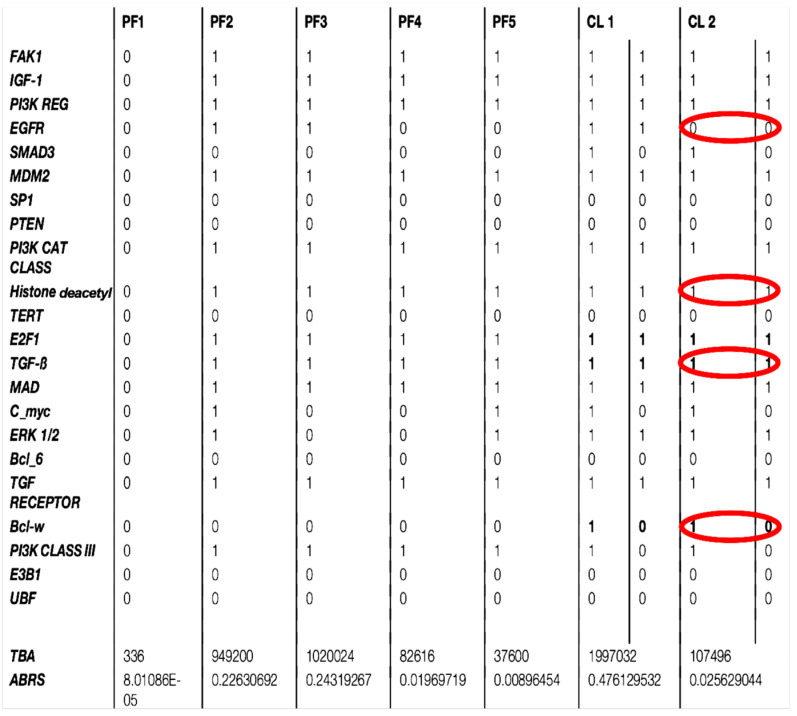
Description of the seven attractors of biliary atresia regulatory network (in parallel updating) with TGFß and Bcl-w increase and EGFR decrease (in red) in pathologic attractor CL2.

The number of attractors can be predicted by an algebraic formula allowing us to calculate the number of attractors of a Boolean network with tangent circuits, as summarized in Figure 6 [60]. In the network of Figure 5, there are two such circuits, one negative of length five and one negative of length eleven. The attractor number predicted by the theory is equal to 7, the same as that obtained from the parallel simulation (using the software Network-Design^©^ [58]) of all trajectories of the biliary atresia network from all possible initial conditions and summarized in Figure 6.

**Figure 7:**
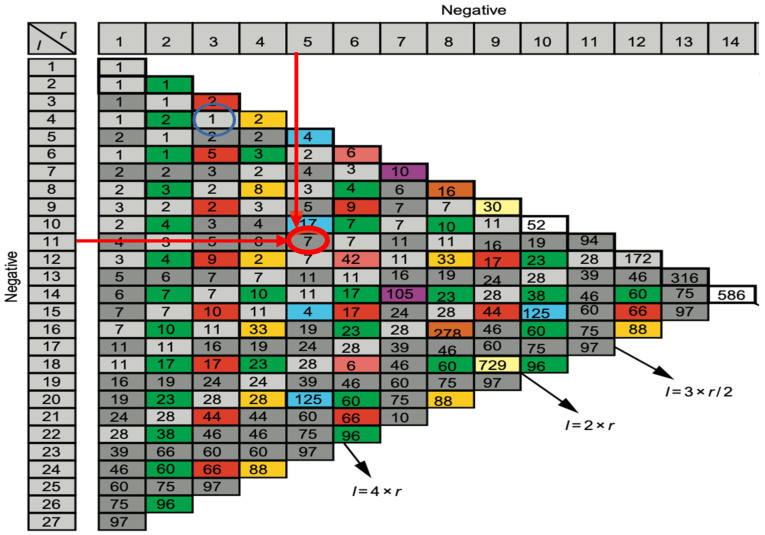
Number of attractors of 2 tangent networks, one negative of length *r* = 5 and the other negative of length *l =* 11.

The attractor limit cycle CL2 of Figure 6 with an attraction basin having an ABRS of 2.6% corresponds to an expression level high for HRS (histone related sequences substrate of HGF), and low for EGFR inhibited by the histone deacetylase [61,62] (Figure 6 in red). Hence, CL2 could correspond to the pathologic attractor BA of Table 1, with a high expression level for HRS causing a low EGFR level, and with high TGFß and Bcl-w levels (in red), both activated by ERK. The fixed points, PF2 and PF3, and the limit cycle CL1 show an increase in EGFR and TGFß with a total ABRS equal to 84.6% and can represent the physiologic behavior of the familial biliary atresia [26,27,28,29,30,31,32,33,34,35,36,37,38,39,40,41,42]. The fixed points PF1, PF4, and PF5 are also characterized by small levels of EGFR and TGFβ (Figure 7), but have small attraction basins with a total ABRS equal to 2.9%. The empiric observation that most of the genes involved in pathologic development have a periodic expression (due in part to the chromatin clock and to the circadian rhythms serving as drivers for the underlying endogenous rhythms) would explain that the pathologic cycle limit CL2 is periodic with an ABRS of 2.6%.

## 3. Discussion

In the previous section, we have presented network dynamics with parallel simulations. Now we intend to discuss simulations corresponding to the two new updating modes we have introduced in Section 2, the intricate and state-dependent ones.

### 3.1. Intricate Updating Mode

In the familial angioedema, some genes are updated systematically together under the control of two groups of genes which are updated alternatively: we have called this updating mode intricate, because it represents a complexification of the classical block-sequential updating mode in which the blocks to update are disjoints.

**Figure 8:**
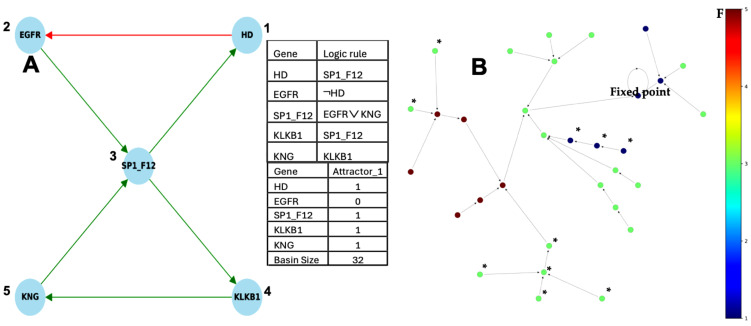
(**A**) Subnework SP1_F12, in which the nodes SP1 and F12 are assumed to be identical; (**B**) global frustration of the network described in (**A**) decreasing along trajectories except for two initial states y_1_ and y_2,_ on the top left in green (global frustration equal to 3), until the fixed point (global frustration equal to 1). The symbol * indicate the states not respecting the decrease of F.

**Figure 9:**
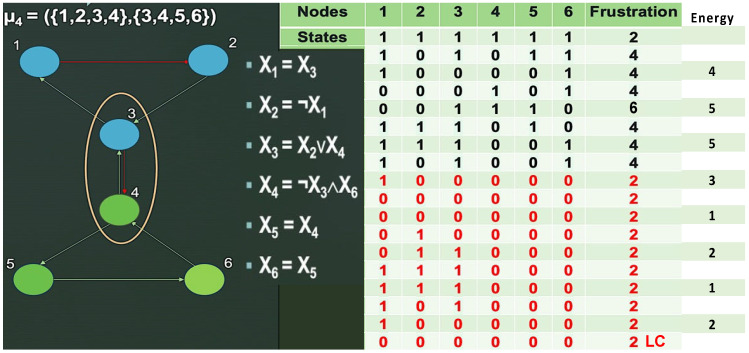
Limit cycle (**right**) from a simulation of the subnetwork SP1 with the interaction graph *G* (**left**) in an intricate block-sequential updating mode (limit cycle LC is shown in red).

For example, we have reduced the networks simulated in parallel in Figure 6 and Figure 8 to a subnetwork whose interaction graph *G* has been described in Figure 1C and Figure 7A. We can define for this subnetwork with graph *G* the intricate updating mode, in which the genes G3 and G4 are always active and the sets of genes G1 and G2 and genes G4 and G5 are alternatively updated in parallel with genes G3 and G4. Figure 9 and Figure S3 (Supplementary Material) correspond to an intricate updating and show a unique limit cycle LC of length 4 with only the physiological states.

Concerning the subnetwork with interaction graph *G*, the difference between the two modes of updating (parallel and intricate) is visible in the case of familial angioedema: in parallel updating, we observe the presence of two limit cycles (Figure 2D), one of which presents a small attraction basin. In the intricate case, we observe only one limit cycle in Figure 9 and Figure S3 of Supplementary Material. Then, we can qualify the subnetwork with graph *G* as not robust for the parallel updating mode, because its number of attractors is reduced after the change in updating mode, but the new attractor obtained is asymptotically stable. The second periodic attractor observed in the parallel mode is unstable because its attraction basin is very small, but it can correspond to a pathologic behavior in the case of familial angioedema.

### 3.2. State-Dependent Updating Mode

**Figure 10:**
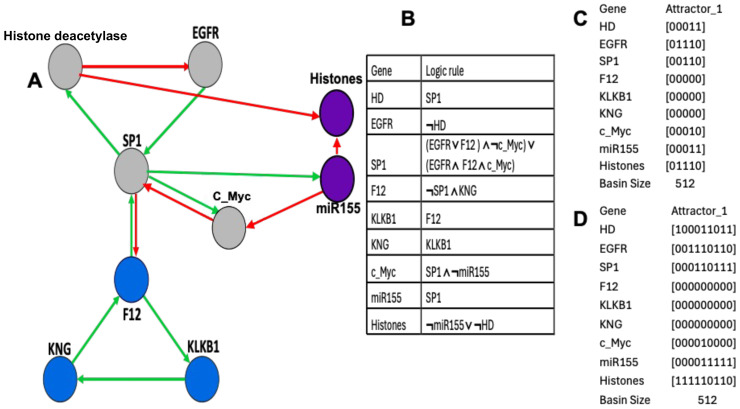
(**A**) Subnetwork SP1 coupled to the histone clock with its regulating microRNA miR155 (partially after [41]). The green color corresponds to activations and the red to inhibitions. (**B**) Logic rule of the SP1 network dynamic. (**C**) The unique attractor limit cycle of length 5 in a parallel simulation. (**D**) The unique attractor limit cycle of length 9 in a state-dependent simulation, i.e., with parallel updating if histones are expressed (state 1) and sequential in order of gene column if histones are not expressed (state 0).

When the updating clock is itself dependent on the state of the genes it updates, the regulation becomes complex and the global behavior of the new network needs the use of a new updating mode, the state-dependent one, as defined in Section 2. It is the case for the network ruling the genes involved in the familial angioedema (Figure **10**).

**Figure 11:**
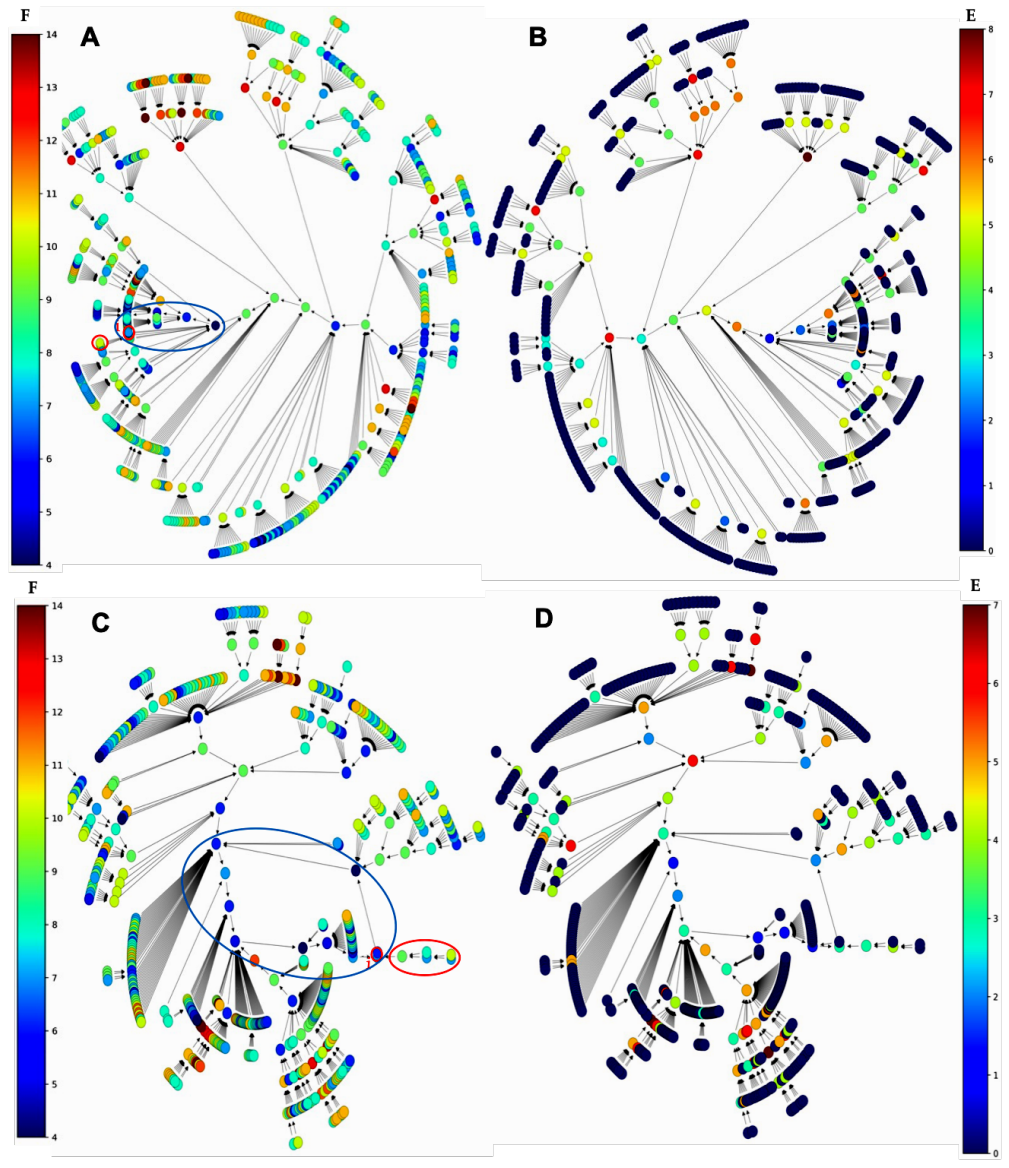
(**A**) Graph of the global frustration of the subnetwork SP1 coupled with histone clock without c_Myc, evolving in parallel from initial conditions to the attractor limit cycle of length 5 (surrounded in blue) and mainly decreasing along trajectories; (**B**) same graph as in (**A**) for the global energy (using the software Espinoza^©^ [59]); (**C**) graph of the global frustration of the subnetwork SP1 coupled with histone clock without c_Myc, evolving in state-dependent updating mode and showing an attractor limit cycle of length 9 (surrounded in blue); (**D**) same graph as in (**C**) for the global energy (using the software Espinoza^©^ [59]). The isochronal basin of the configuration i is indicated in red on graphs (**A**,**C**).

The SP1 subnetwork (Figure 10A) is activated in two different ways, with the same logic transition rule (Figure 10B):

(i) A simulation is performed with a classical parallel updating mode and shown the presence of a single attractor, a limit cycle of length 5. Figure 11A (respectively, Figure 11B) shows the frustration (resp. energy) function on the trajectories. The basin of attraction of the configuration i of the limit cycle in Figure 11A, called the isochronal basin of i, is limited to five initial configurations rotating in phase with i on the limit cycle after one iteration.
(ii) the second simulation was performed with the state-dependent updating mode, i.e., in parallel mode if histone genes were expressed (state 1) and sequential otherwise (in the order of the genes in Gene column of Figure 10B).

This second simulation showed the presence of a single attractor, a limit cycle of length 9, repeating the three central configurations of the previous limit cycle with different isochronal basins but of the same order of magnitude, for example, four (resp. five) initial configurations for the basin of the configuration i of the limit cycle in Figure 10A (resp. Figure 10C). The biliary atresia network coupled to the chromatin clock network is therefore robust, both for changes (i) in initial conditions, since in each simulation case only one attractor limit cycle is observed, and (ii) in structure, whether in case of parallel or state-dependent updating modes, and for the addition of a new subnetwork.

### 3.3. Comparison Between Updating Modes of the Subnetwork SP1

With regard to the differences observed between the updating modes of the subnetwork SP1, Figure 9 shows, for the intricate mode, a single limit cycle attractor of length 8 whose basin of attraction is represented by all states except the attractant ones. In the Figure S1 (Supplementary Material), we observe two limit cycles for the parallel mode, one of length 2 with an attraction basin of size 2 and the second of length 6 with an attraction basin of size 54 on Figure S2 (Supplementary Material). Figure 11 clearly shows the difference between the parallel mode and the state-dependent updating mode of the subnetwork SP1 coupled with histone clock without c_Myc: in the first case, we observe a limit cycle attractor of length 5 with isochronous basins for the attractant states of approximately the same size. In the second case, we observe a limit cycle attractor of length 9 with isochronous basins for the attractant states of very different sizes.

## 4. Material and Methods

Data about the gene interactions have been founded in dedicated databases [60,63,64,65] and simulations of Boolean networks were carried out with the public software BoolNet [57] and by specific programs developed in the authors’ laboratories [58,59]. Theoretical results come from previous work [53,54,55,56,66,67,68,69,70,71,72,73,74,75,76,77,78,79]. They aim to clarify important concepts such as Boolean network, interaction graph, attractor, and basin of attraction.

### 4.1. Boolean Networks

A Boolean network is just a process marking the vertices states of a network as ‘on/off’, with several ways to update these states [80,81,82]. Theoretically, we denote the whole configuration of a Boolean network N of size n at time t by x(t) = {xi(t)}_i=1,n_, where x(t) ∈ Ω= {0,1}^n^, the set of all possible Boolean configurations of N. Each node i of N represents the ith gene denoted G_i_ and is connected to k(i) other genes i_1_, …, i_k(i)_ (possibly equal to i itself). The state x_i_ of G_i_ is updated according to a specific rule:

x_i_(t + 1) = F_i_(x(t)) (1)

where F_i_ is a Boolean transition function and x_i_(t + 1) represents the state of the gene G_i_ at time (t + 1), equal to 1 if G_i_ is expressed and 0 if not. The Boolean function F_i_ is represented by a logic equation which gives the state of G_i_ as a function of its connected neighbors. For example, if n = 6 (as in Figure 1C), this logic equation can be

F_1_ = G_3_, F_2_ = −G_1_, F_3_ = G_2_, F_4_ = −G_3_ ∧ G_6_, F_5_ = G_4_, F_6_ = G_5_ (2)

The logic formalism in the above example is equivalent to a Hopfield transition function with weights 1 (for an activation) or −1 (for an inhibition), a temperature 0 and a threshold 0:

x_4_(t + 1) = H(−x_3_(t) + x_6_(t)), (3)

where H is the Heaviside function (H(y) = 0 if y ≤ 0 and H(y) = 1 if y > 0).

### 4.2. Interaction Graph

The interaction graph *G*(N) of the network N is represented by an oriented graph in which the n nodes correspond to genes and directed edges to the interactions between these genes. Each interaction is characterized by an influence weight w*_ij_*, which represents the influence that the gene j node of the interaction graph *G*(N) exerts on the gene i, through the influence of its messenger RNA or the protein the gene j expresses: w*_ij_* = 1 (respectively, w*_ij_* = −1) corresponds to the sign + or the color green (respectively, the sign—or the color red) on the edge between j and i, if j activates (respectively, inhibits) the gene i. If j has no influence on i, there is no edge, no sign, and no color between nodes j and i.

### 4.3. Attractors

Since R. Thom’s work [66], definitions of attractors are numerous, but we give a version available both for continuous and discrete cases [67]. First we define the Birkhoff limit set L(x) of an initial condition x in the state space W as the set of accumulation points of a trajectory T, where T(x,t) is the state reached at time t from the initial condition x: L(x) = {y∈W; ∀ε > 0, ∀t > 0, ∃s > t/d(T(x,s),y) ≤ ε}. The attraction basin B(A) of a subset A of W is the set of initial conditions x with L(x)⊂A and ABRS(A) = card(B(A))/2^n^ is its relative size. A is an attractor if it verifies the following:

(i) A is a fixed set for the composed set operator LoB: A = L(B(A));
(ii) there is no B ≠ A, B⊃**A** and verifying (i), where **A** is A with shadow trajectories [53];
(iii) there is no C ≠ A, C⊂A and verifying (i) and (ii).

In the Boolean case, under the influence of the transition functions F_i_, the configuration x(t) of the network can evolve in Ω toward two possible asymptotic behaviors called attractors: a fixed configuration (also called fixed point) or a cycle of configuration (also called limit cycle). One of the challenges in a genetic regulation network is to determine the nature of its attractors (fixed point or limit cycle), which can have a great influence on its physiological or pathological character.

For each attractor, we call the attraction basin the set of all initial configurations of the network which lead to it after a finite number of iterations. In applications, we determine the possible attractors and their attraction basins (at least their size). Historically, the nature of attractors has been introduced by Poincaré [54], the problem of their number by Hilbert [55] and their use in biological regulation has been proposed by Delbrück [56], Kauffman [68], and Thomas [69,70].

**Figure 12:**
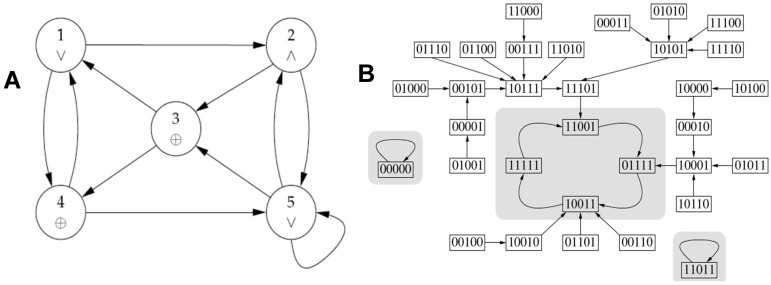
(**A**) Boolean network with Boolean functions inside the vertices; (**B**) Trajectories of the Boolean network parallelly updated with indication of the 3 attractors (in gray) towards which the trajectories converge.

By considering the Boolean network of Figure 12A, in which the Boolean functions are represented by the logic operators inside the vertices, we see its 3 attractors on Figure 12B.

### 4.4. Updating Modes

The updating mode of a Boolean network is a rule describing in which order the state of the nodes has to be updated. There are two classical types of updating modes:

- the block-sequential mode, which consists of choosing a partition of N in m disjoint subsets of nodes S1, …, Sm, with ∪k=1,m Sk = N, which are updated sequentially, the nodes of each subset being updated parallelly. If each subset is a singleton, the updating mode is called sequential, the choice of the order being possibly random.
- the block-parallel mode, which consists of choosing a partition of N in m disjoint subsets, which are updated parallelly, the nodes of each subset being updated sequentially. If the partition has only a set, the updating mode is called parallel.

We now introduce two new updating modes:

- the block-intricate sequential (respectively, parallel) mode, if the subsets of the partition of N are not obligatory disjoint, i.e., if there exists two indices i and j in {1, …, m}, such as Si ∩ Sj = ∅. The subsets Sk, k∈{1, …, m}, are updated sequentially (respectively, parallelly), nodes of each subset being updated parallelly (respectively, sequentially).
- the state-dependent mode, in which the mode of iteration can be chosen at each iteration of the network N depending on the state of nodes of a subset C of N, considered as a clock, e.g., if the nodes of C are in state 0, then the updating mode is sequential inside the subsets Sk, and if they are in state 1, the updating mode is parallel.

### 4.5. Different Notions of Robustness

The network’s robustness is the network’s ability to resist interference. Mathematically speaking, a network is robust if its attractors do not vary in case of five perturbation types: initial condition, parameter value, interaction graph, transition function or updating mode.

Corresponding to these perturbations, there are three types of robustness:

- Trajectorial robustness, which corresponds to the existence of a distance threshold respected between original and perturbed trajectories;
- Asymptotic robustness, which corresponds to the conservation of the attractor of any trajectory after a perturbation, even if the transient part of this trajectory is modified;
- Structural robustness, which corresponds to the conservation of the number and nature of the attractors in response to a structural perturbation (change in interaction graph, transition function or updating clock).

### 4.6. Frustration, Energy and Entropy

The local kinetic energy E_i_(x(t)) between t and (t + 1) is defined, because x_i_ = 1 or 0, by

E_i_(x(t)) = [(x_i_(t + 1) − x_i_(t))/(t + 1 − t)]^2^/2 = |x_i_ (t + 1) – x_i_(t)|/2 (4)

The global kinetic energy of x, E(x(t)), is the sum of the local energies over the nodes of N.

F(x(t)), the global frustration of x, is the sum of the local frustrations F_ij_(x(t)), i.e., the number of pairs (i,j) where the values of x_i_ and x_j_ at time t are contradictory with the sign w_ij_ of the interaction between genes j and i:

F(x(t)) = Σ_i,j∈{1,n}_F_ij_(x(t)) (5)

where F_ij_ is the local frustration of the pair of nodes (i,j) defined by

F_ij_(x) = 1, if w_ij_ = 1 (resp. −1), x_j_ = 1, x_i_ = 0 (resp. 1), or x_j_ = 0, x_i_ = 1 (resp. 0). F_ij_(x) = 0 elsewhere (6)

The general formula of the local frustrations for edge ij and node i can be written as

F_ij_(x) = ½ + w_ij_(|x_j_ − x_i_| − ½) and F_i_(x) = S_j=1,n_ w_ij_ F_ij_(x) (7)

We can calculate the global frustration along a circuit by summing all its local frustrations.

If w_ij_ = 1, F_ij_(x(t)) =|x_j_(t) − x_i_(t)| = |x_i_(t + 1) − x_i_(t)|= 2E_i_(x(t)).

If w_ij_ = −1, F_ij_(x(t)) = 1 − |x_j_ (t) − x_i_(t)| = 1 − |1 − x_i_(t + 1) − x_i_(t)|= 2E_i_(x(t)).

Then, on a circuit without self-interaction, F(x) = E(x). If the logic transition function is F_1_ = G_3_, F_2_ = −G_1_, F_3_ = G_2_vG_5_, F_4_ = G_3_, F_5_ = G_4_ as for the network of Figure 8A, there exists a unique attractor, the fixed point (10111), whose ABRS = 100%. Its global frustration is decreasing along trajectories except for the initial configurations marked with * on Figure 8B.

## 5. Conclusions

In this paper, we studied three examples of classic and new updating modes applied to familial diseases, familial angioedema, osteogenesis imperfecta and biliary atresia, whose genetic regulation networks are, in general, updated using the block-sequential updating mode. In certain cases, as in familial angioedema, it is more convenient to use new updating modes, the intricate block-sequential mode, in which the nodes of the Boolean automata network are updated differently, with some being updated at each iteration and others at lower frequencies [72], and the state-dependent updating mode, in which the state of certain genes to update can influence the genes governing their updating schedule.

Indeed, in the application of Boolean networks to genetic regulation, the absence of precise information on the mode of updating the state of the genes (i.e., the expression of the genes involved in the network), it is necessary to examine the consequences of a change in mode on the dynamics of the network, which can be a modification of the number, nature, and size of the basins of attraction of its attractors. If the characteristics of the attractors are invariant under a change in updating mode, the network is said to be structurally robust, but if not, it is said structurally unstable. Therefore, in this last case, the choice of the most realistic updating mode of the network dynamics is crucial regarding the practical consequences of the discrete modeling of the gene expression, in order to explain the genesis of a familial pathology involving well-identified interacting genes, which is the case in the diseases taken as examples in the present study (namely familial angioedema, osteogenesis imperfecta and biliary atresia). Such an approach is particularly useful for identifying changes in critical genes and gene interactions at the origin of the disease.

In the perspective of this work, given a connected Boolean network with n nodes, m edges, and a configuration x ∈ {0,1}^n^ = Ω, the state space of its dynamics and the calculations presented in this manuscript reveal a generally easy-to-check property: the frustration of x is a unique integer k(x), with 0 ≤ k(x) ≤ m. However, if we define TF as the total frustration of the network—corresponding to the sum of the frustrations across all its 2^n^ configurations—it is easy to prove that this value is always strictly greater than 0 (since there will always be at least one configuration with one frustrated edge) and strictly less than m2^n^ (as there will always be at least one configuration with at least one unfrustrated edge). That is, 0 < TF < m2^n^. In this context, although a completely frustration-free dynamic is not possible, it may be useful for modeling certain phenomena of interest to consider, in future works, networks with very low total frustration as the best representatives of the observed regulations [83,84,85,86,87]. For example, in a network with only activations between its genes, the introduction of a microRNA-type inhibitor on one of its genes suppresses frustrations. This is the case in a gene circuit without inhibition: introducing an inhibitor external to the circuit on one of its genes causes the appearance of a single attractor corresponding to all genes in the zero state, i.e., a configuration without frustration. This is also the case in Figure 3D, where the introduction of the miR200 inhibitor diminishes the frustration: the gene Col1A1 passes from a frustration of 4 to 0, SP1 from 8 to 9, Smad3 from 10 to 0, TGFß from 18 to 9, Notch1 from 4 to 13, CCND1 from 0 to 8, and Pax8 from 4 to 12 that is, globally, from a mean frustration of attracting state of 6 with miR200 to 5,66 without.

In conclusion, the scope of application of the new update rules is broad, and other Boolean networks, such as multilayer networks using the AlexNet ReLu sequential block-wise update rule [88] involved in multisensor detection, could use the new concepts presented in this paper, namely the notions of frustration and of intricate and state-dependent updating, to more closely resemble the human detection processes they simulate.

## Figures and Tables

**Table 1:** Pathologic (BA) expression with TGFß, Bcl-w and HGF increase causing EGFR decrease (circles in red).

Transcript Identity	Intensity Normal	Intensity BA	Ratio BA/Normal
Bcl-w	16.7	28.8	1.7
Laminin BP (binding protein)	0.7	7.7	11
HRS (HGF-regulated tyrosine kinase substrate)	0.5	3.5	6.9
Thymosin ß4	0.6	4.1	6.5
Thymosin ß10	0.5	2.5	5.4
TGF-ß	0.6	2.1	3.3
TIMP-1 (tissue inhibitor of metalloproteinase)	1.8	3.9	2.2
SRP4 (signal recognition protein)	2	7.1	3.6
SRP9	1.7	5.6	3.3
SNAP 45 (soluble NSF attachment protein)	1.2	5.6	4.5
Alu RNA BP	0.8	6.9	8.5
Supt5h (human homologue of yeast transcription factor SPT5)	1.9	4.5	2.3
Elf-2α kinase	4.5	12.1	2.7
HSP 27 (heat shock protein)	2.1	3.7	1.8

## Data Availability

The original contributions presented in this study are included in the article/Supplementary Materials. Further inquiries can be directed to the corresponding author.

## Supplementary Materials

The following supporting information can be downloaded at: https://www.mdpi.com/article/10.3390/ijms262411976/s1.
