# Supplementary material for: Boolean Networks with Classic and New Updating Modes Applied to Genetic Regulation in Some Familial Diseases
Source: Int J Mol Sci. 2025 Dec 12;26(24):11976. doi: 10.3390/ijms262411976 (PMC12732346; doi:10.3390/ijms262411976)
Supplement: Supplementary file 1 [file ijms-26-11976-s001.zip › ijms-3910501-supplementary.pdf]

Supplementary material
Boolean networks with parallel and intricate updating modes

Figure S1. Simulations in parallel updating mode of the subnetwork SP1 regulating the gene expression in angioedema (using the softwares BoolNet© and Espinoza©). They show two limit-cycles LC1 and LC2 (in red) as attractors.

Nodes	1	2	3	4	5	6	Frustration	Energy
States	1	1	1	1	1	1	2	
	1	0	1	0	1	1	4	2
	1	0	0	0	0	1	4	2
	0	0	0	1	0	0	4	3
	0	1	1	0	1	0	4	4
	1	1	1	0	0	1	4	3
	1	0	1	0	0	0	2	2 LC1

Nodes	1	2	3	4	5	6	Frustration	Energy
States	0	0	0	1	1	1	2	
	0	1	1	1	1	1	2	2
	1	1	1	0	1	1	4	2
	1	0	1	0	0	1	4	2 LC1

Nodes	1	2	3	4	5	6	Frustration	Energy
States	0	0	0	0	1	0	4	
	0	1	0	0	0	1	4	3
	0	1	1	1	0	0	4	3 LC1

Nodes	1	2	3	4	5	6	Frustration	Energy
States	0	0	0	1	1	0	4	
	0	1	1	0	1	1	4	4
	1	1	1	0	0	1	4	2 LC1

Nodes	1	2	3	4	5	6	Frustration	Energy
States	1	1	0	1	0	0	6	
	0	0	1	0	1	0	6	5
	1	1	0	0	0	1	6	5
	0	0	1	1	0	0	6	5 LC1

Nodes	1	2	3	4	5	6	Frustration	Energy
States	1	1	0	1	1	1	4	
	0	0	1	1	1	1	4	3 LC1

Nodes	1	2	3	4	5	6	Frustration	Energy
States	1	1	1	1	1	0	4	
	1	0	1	0	1	1	4	3 LC1

Nodes	1	2	3	4	5	6	Frustration	Energy
States	1	1	0	0	1	0	6	
	0	0	1	0	0	1	6	5
	1	1	0	0	0	0	6	4
	0	0	1	0	0	0	4	3 LC2

Nodes	1	2	3	4	5	6	Frustration	Energy
States	1	1	0	1	0	0	6	
	0	0	1	0	1	0	6	5 LC1

Nodes	1	2	3	4	5	6	Frustration	Energy
States	1	1	0	0	1	0	6	
	0	0	1	0	0	1	6	5
	1	1	0	0	0	0	4	4 LC2

Nodes	1	2	3	4	5	6	Frustration	Energy
States	1	0	0	0	0	0	2	LC1

Nodes	1	2	3	4	5	6	Frustration	Energy
States	1	0	0	0	0	1	4	
	0	0	0	1	0	0	4	3 LC1

Nodes	1	2	3	4	5	6	Frustration	Energy
States	0	1	0	1	1	1	2	
	0	1	1	1	1	1	2	1 LC1

Nodes	1	2	3	4	5	6	Frustration	Energy
States	0	0	0	1	0	0	4	
	0	1	1	0	1	0	4	4 LC1

Nodes	1	2	3	4	5	6	Frustration	Energy
States	0	0	0	0	1	1	4	
	0	1	0	1	0	1	4	3
	0	1	1	1	1	0	4	3
	1	1	1	0	1	1	4	3
	1	0	1	0	0	1	4	2 LC1

Nodes	1	2	3	4	5	6	Frustration	Energy
States	0	0	0	0	0	1	4	
	0	1	0	1	0	0	4	3
	0	1	1	0	1	0	4	3
	1	1	1	0	0	1	4	3
	1	0	1	0	0	0	2	2
	1	0	0	0	0	0	2	1
	0	0	0	0	0	0	2	1
	0	1	0	0	0	0	2	1 LC1

Nodes	1	2	3	4	5	6	Frustration	Energy
States	0	0	0	1	0	1	4	
	0	1	1	1	1	0	4	4 LC1

Nodes	1	2	3	4	5	6	Frustration	Energy
States	1	0	0	1	1	1	2	
	0	0	1	1	1	1	4	2
	1	1	1	0	1	1	4	3
	1	0	1	0	0	1	4	2 LC1

Nodes	1	2	3	4	5	6	Frustration	Energy
States	1	1	0	0	1	1	6	
	0	0	1	1	0	1	6	5
	1	1	1	0	1	0	4	5
	1	0	1	0	0	1	4	3 LC1

Nodes	1	2	3	4	5	6	Frustration	Energy
States	1	1	1	1	0	1	4	
	1	0	1	0	1	0	4	4
	1	0	0	0	0	1	4	3
	0	0	0	1	0	0	4	3 LC1

Nodes	1	2	3	4	5	6	Frustration	Energy
States	1	1	1	0	1	0	4	
	1	0	1	0	0	1	4	3 LC1

Nodes	1	2	3	4	5	6	Frustration	Energy
States	1	0	1	0	0	0	2	
	1	0	0	0	0	0	2	1 LC1

Nodes	1	2	3	4	5	6	Frustration	Energy
States	1	0	0	0	1	0	4	
	0	0	0	0	0	1	4	3 LC1

Nodes	1	2	3	4	5	6	Frustration	Energy
States	0	0	1	0	0	0	4	
	1	1	0	0	0	0	4	3
	0	0	1	0	0	0	4	3 LC2

Nodes	1	2	3	4	5	6	Frustration	Energy
States	0	0	0	0	0	1	4	
	0	1	0	1	0	0	4	3 LC1

Nodes	1	2	3	4	5	6	Frustration	Energy
States	1	1	1	1	0	0	4	
	1	0	1	0	1	0	4	3 LC1

Nodes	1	2	3	4	5	6	Frustration	Energy
States	1	1	0	0	0	1	6	
	0	0	1	1	0	0	6	5
	1	1	1	0	1	0	4	4
	1	0	1	0	0	1	4	3 LC1

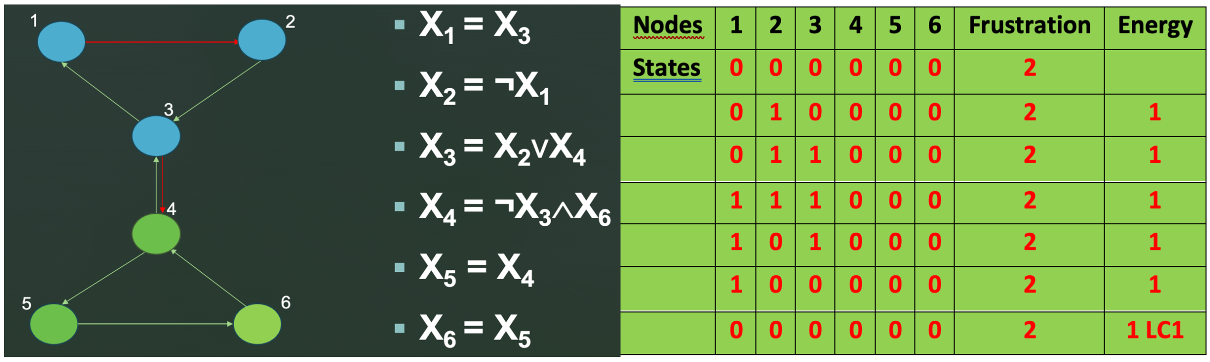

Nodes	1	2	3	4	5	6	Frustration	Energy
States	0	0	0	0	0	0	2	
	0	1	0	0	0	0	2	1
	0	1	1	0	0	0	2	1
	1	1	1	0	0	0	2	1
	1	0	1	0	0	0	2	1
	1	0	0	0	0	0	2	1
	0	0	0	0	0	0	2	1 LC1

Figure S2. Attractor limit cycle LC1 from the simulation of the dynamics of the subnetwork SP1 regulating the gene expression in angioedema (parallel updating).

Figure S3. Simulations of the subnetwork SP1 with the intricate block-sequential updating mode (using the softwares BoolNet© [57] and Espinoza® [59]), showing only the limit-cycle LC.

Nodes	1	2	3	4	5	6	Frustration	Energy
States	0	0	0	0	0	0	2	
	0	1	0	0	0	0	2	
	0	1	1	0	0	0	2	2
	1	1	1	0	0	0	2 LC	

Nodes	1	2	3	4	5	6	Frustration	Energy
States	0	0	0	1	1	1	2	
	0	1	1	1	1	1	2	
	0	1	1	0	1	1	2	3
	1	1	1	0	1	1	4	
	1	1	1	0	0	1	2 LC	2

Nodes	1	2	3	4	5	6	Frustration	Energy
States	0	0	0	1	0	0	4	
	0	1	1	0	0	0	2	
	0	1	1	0	0	0	2	3
	1	1	1	0	0	0	2 LC	

Nodes	1	2	3	4	5	6	Frustration	Energy
States	0	0	0	1	1	0	4	
	0	1	1	0	1	0	4	
	0	1	1	0	0	1	4	5
	1	1	1	0	0	1	4	
	1	1	1	0	0	0	2 LC	2

Nodes	1	2	3	4	5	6	Frustration	Energy
States	0	1	0	1	1	1	2	
	0	1	1	1	1	1	2	
	0	1	1	0	1	1	4	2
	1	1	1	0	1	1	4	
	1	1	1	0	0	1	4 LC	2

Nodes	1	2	3	4	5	6	Frustration	Energy
States	1	1	1	1	1	0	4	
	1	0	1	0	1	0	4	
	1	0	0	0	0	1	4 LC	5

Nodes	1	2	3	4	5	6	Frustration	Energy
States	1	1	1	0	1	0	4	
	1	0	1	0	1	0	4	
	1	0	0	0	0	1	4 LC	4

Nodes	1	2	3	4	5	6	Frustration	Energy
States	1	1	0	0	1	0	6	
	0	0	1	0	1	0	6	
	0	0	0	0	0	1	4	4
	0	1	0	1	0	1	4	
	0	1	1	1	1	0	4 LC	5

Nodes	1	2	3	4	5	6	Frustration	Energy
States	1	1	0	0	0	0	4	
	0	0	1	0	0	0	4	
	0	0	0	0	0	0	2 LC	2

Nodes	1	2	3	4	5	6	Frustration	Energy
States	1	0	0	0	0	0	2	
	0	0	0	0	0	0	2 LC	

Nodes	1	2	3	4	5	6	Frustration	Energy
States	1	0	0	0	0	1	4	
	0	0	0	1	0	1	4	
	0	0	1	1	1	0	6 LC	5

Nodes	1	2	3	4	5	6	Frustration	Energy
States	0	0	1	0	0	0	4	
	1	1	0	0	0	0	4	
	1	1	1	0	0	0	2 LC	2

Nodes	1	2	3	4	5	6	Frustration	Energy
States	0	0	0	0	1	1	4	
	0	1	0	1	1	1	2	
	0	1	1	1	1	1	2	3
	1	1	1	0	1	1	4	
	1	1	1	0	0	1	4 LC	3

Nodes	1	2	3	4	5	6	Frustration	Energy
States	0	0	0	0	0	1	4	
	0	1	0	1	0	1	4	
	0	1	1	1	1	0	4	5
	1	1	1	0	1	0	4 LC	

Nodes	1	2	3	4	5	6	Frustration	Energy
States	0	0	0	1	0	1	4	
	0	1	1	1	0	1	4	
	0	1	1	0	1	0	4	5
	1	1	1	0	1	0	4	
	1	0	1	0	0	1	4 LC	4

Nodes	1	2	3	4	5	6	Frustration	Energy
States	0	1	1	1	1	1	2	
	1	1	1	0	1	1	4 LC	

Nodes	1	2	3	4	5	6	Frustration	Energy
States	0	0	0	0	1	0	4	
	0	1	0	0	1	0	4	
	0	1	1	1	0	1	4 LC	5

Nodes	1	2	3	4	5	6	Frustration	Energy
States	0	0	1	1	1	1	4	
	1	1	1	0	1	1	4 LC	

Nodes	1	2	3	4	5	6	Frustration	Energy
States	1	0	0	1	1	1	2	
	0	0	1	1	1	1	4	
	0	0	1	0	1	1	4	3
	1	1	0	0	1	1	6	
	1	1	1	1	0	1	4	4
	1	0	1	0	0	1	4 LC	

Nodes	1	2	3	4	5	6	Frustration	Energy
States	1	1	0	1	1	1	4	
	0	0	1	1	1	1	4 LC	

Nodes	1	2	3	4	5	6	Frustration	Energy
States	1	1	0	0	1	1	6	
	0	0	1	1	1	1	4 LC	

Nodes	1	2	3	4	5	6	Frustration	Energy
States	1	1	1	1	0	1	4	
	1	0	1	0	0	1	4 LC	

Nodes	1	2	3	4	5	6	Frustration	Energy
States	1	1	0	1	0	0	6	
	0	0	1	0	0	0	4	
	0	0	0	0	0	0	2 LC	3

Nodes	1	2	3	4	5	6	Frustration	Energy
States	1	0	1	0	0	0	2	
	1	0	0	0	0	0	2	
	1	0	0	0	0	0	2	1
	0	0	0	0	0	0	2 LC	

Nodes	1	2	3	4	5	6	Frustration	Energy
States	1	1	1	1	0	0	4	
	1	0	1	0	0	0	2 LC	

Nodes	1	2	3	4	5	6	Frustration	Energy
States	1	0	0	0	1	0	4	
	0	0	0	0	1	0	4	
	0	0	0	0	0	1	4 LC	3

Comparison between updating modes of the subnetwork SP1

With regard to the differences observed between the updating modes of the subnetwork SP1, Figure 8 shows, for the intricate mode, a single limit cycle attractor of length 8 whose basin of attraction is represented by all states except the attractant ones. In the Supplementary material, we observe two limit cycles for the parallel mode, one of length 2 with an attraction basin of size 2 and the second of length 6 with an attraction basin of size 54.
